# A novel culture medium with reduced nutrient concentrations supports the development and viability of mouse embryos

**DOI:** 10.1038/s41598-020-66019-4

**Published:** 2020-06-09

**Authors:** Alison F. Ermisch, Jason R. Herrick, Rolando Pasquariello, McKenna C. Dyer, Sarah M. Lyons, Corey D. Broeckling, Sandeep K. Rajput, William B. Schoolcraft, Rebecca L. Krisher

**Affiliations:** 1 Colorado Center for Reproductive Medicine, Lone Tree, CO USA; 2 Colorado State University, Fort Collins, CO USA; 3 Present Address: University of Nebraska-Lincoln, Lincoln, NE USA; 4 Present Address: Omaha’s Henry Doorly Zoo & Aquarium, Omaha, NE USA; 5 Present Address: University of Milan, Milan, Italy

**Keywords:** Embryology, Infertility, Translational research

## Abstract

Further refinement of culture media is needed to improve the quality of embryos generated *in vitro*. Previous results from our laboratory demonstrated that uptake of nutrients by the embryo is significantly less than what is supplied in traditional culture media. Our objective was to determine the impact of reduced nutrient concentrations in culture medium on mouse embryo development, metabolism, and quality as a possible platform for next generation medium formulation. Concentrations of carbohydrates, amino acids, and vitamins could be reduced by 50% with no detrimental effects, but blastocyst development was impaired at 25% of standard nutrient provision (reduced nutrient medium; RN). Addition of pyruvate and L-lactate (+PL) to RN at 50% of standard concentrations restored blastocyst development, hatching, and cell number. In addition, blastocysts produced in RN + PL contained more ICM cells and ATP than blastocysts cultured in our control (100% nutrient) medium; however, metabolic activity was altered. Similarly, embryos produced in the RN medium with elevated (50% control) concentrations of pyruvate and lactate in the first step medium and EAA and Glu in the second step medium were competent to implant and develop into fetuses at a similar rate as embryos produced in the control medium. This novel approach to culture medium formulation could help define the optimal nutrient requirements of embryos in culture and provide a means of shifting metabolic activity towards the utilization of specific metabolic pathways that may be beneficial for embryo viability.

## Introduction

In the United States, implantation and live birth rates in clinical ART cycles remain low. According to the National ART Summary^1^, the percentage of *in vitro* produced human embryos capable of implantation is less than 42%, even in women age 35 or younger. These statistics clearly demonstrate a need for improved embryo culture media that produce high quality embryos capable of implantation, maintenance of pregnancy, and development into healthy offspring. With the shift in human IVF towards blastocyst transfer and the associated increase in the time embryos spend *in vitro,* the need for media capable of supporting the development of high quality blastocysts has become essential^2,3^. Currently, there are two prevailing theories regarding the composition of culture media to support blastocyst development. Use of a single culture medium from the one cell to blastocyst stage is largely based on the work of Biggers *et al*. and the formulation of potassium simplex optimized medium (KSOM; Table 1)^4,5^. Utilization of this and other similar media is based on the theory that providing the embryo with all of the nutrients it may require throughout preimplantation development allows the embryo to ‘choose’ which nutrients it uses and when. Conversely, sequential culture systems, notably the G1/2 series (Tables 1 and 2) developed by Gardner *et al*.^6,7^, use two media to mimic known differences in the concentrations of nutrients present in the oviductal and uterine environments^8,9^. The extensive influence of KSOM and G1/2 on the composition of clinical embryo culture media is evident, with most commercial media closely resembling, if not replicating, published formulations of these two media^10–13^. Similarities between KSOM and most of the commercially available “single step” media are particularly striking^11^. However, clinical trials comparing culture media have yielded conflicting results and the identification of an optimum culture system to support the development of high-quality human blastocysts has proven almost impossible^14–16^. Further optimization of culture conditions for human embryos, and indeed those of other species as well, may require a novel approach for medium formulation.

**Table 1:** Nutrient concentrations (mM) in various dilutions (100 to 25%) of Optimized Embryo Culture Medium 1 (OEC1, 0–48 h of culture), as well as those reported for murine oviductal fluid and other media used for murine embryo culture.

	OEC1				RN1+PL	*In Vivo*Oviduct	Whitten	KSOMAA	G1.2
	100%	75%	50%	25%					
Glucose	0.500	0.375	0.250	0.125	0.125	1.09	5.56	0.2	0.5
Pyruvate	0.300	0.225	0.150	0.075	***0.150***	0.37	0.33	0.2	0.32
L-Lactate	10.000	7.500	5.000	2.500	***5.000***	10.90	25	10	10.5
Citrate	0.500	0.375	0.250	0.125	0.125		0	0	0.5
Ala-Gln	0.500	0.375	0.250	0.125	0.125	1.44	0	1	0.5
Taurine	0.100	0.075	0.050	0.025	0.025	6.64	0	0	0.1
EDTA	0.010	0.008	0.005	0.003	0.003				
**NEAA**									
Ala	0.100	0.075	0.050	0.025	0.025	2.52	0	0.05	0.1
Asn	0.100	0.075	0.050	0.025	0.025	0.23	0	0.05	0.1
Asp	0.100	0.075	0.050	0.025	0.025	0.94	0	0.05	0.1
Glu	0.100	0.075	0.050	0.025	0.025	1.37	0	0.05	0.1
Gly	0.100	0.075	0.050	0.025	0.025	3.22	0	0.05	0.1
Pro	0.100	0.075	0.050	0.025	0.025		0	0.05	0.1
Ser	0.100	0.075	0.050	0.025	0.025	0.59	0	0.05	0.1
**EAA**									
Arg	0.150	0.113	0.075	0.038	0.038	0.05	0	0.3	0
Cys	0.025	0.019	0.013	0.006	0.006		0	0.05	0
His	0.050	0.038	0.025	0.013	0.013	0.18	0	0.1	0
Iso	0.100	0.075	0.050	0.025	0.025	0.20	0	0.2	0
Leu	0.100	0.075	0.050	0.025	0.025	0.38	0	0.2	0
Lys	0.100	0.075	0.050	0.025	0.025	0.26	0	0.2	0
Met	0.025	0.019	0.013	0.006	0.006	0.17	0	0.05	0
Phe	0.050	0.038	0.025	0.013	0.013	0.20	0	0.1	0
Thr	0.100	0.075	0.050	0.025	0.025	0.80	0	0.2	0
Trp	0.013	0.009	0.006	0.003	0.003	0.05	0	0.025	0
Tyr	0.050	0.038	0.025	0.013	0.013	0.26	0	0.1	0
Val	0.100	0.075	0.050	0.025	0.025	0.35	0	0.2	0
Total =	**13.47**	**10.10**	**6.74**	**3.37**	**5.94**	**32.21**	**30.89**	**13.48**	**13.12**
(mmol nutrients/L)									
Reference						9	46	13	12

**Table 2:** Nutrient concentrations (mM) in various dilutions (100 to 25%) of Optimized Embryo Culture Medium 2 (OEC2, 48–112 h of culture), as well as those reported for murine uterine fluid and other media used for murine embryo culture.

	OEC2				RN2+PL	RN2 + EAA + Glu	*In Vivo*Uterus	Whitten	KSOMAA	G2.2
	100%	75%	50%	25%						
Glucose	3.000	2.250	1.500	0.750	0.750	0.750	0.61	5.56	0.2	3.15
Pyruvate	0.100	0.075	0.050	0.025	***0.050***	0.025	0.25	0.33	0.2	0.1
L-Lactate	6.000	4.500	3.000	1.500	***3.000***	1.500	9.40	25	10	5.87
Citrate	0.500	0.375	0.250	0.125	0.125	0.125		0	0	0.5
myo-Insoitol	0.086	0.065	0.043	0.022	0.022	0.022		0	0	0.01
Ala-Gln	1.000	0.750	0.500	0.250	0.250	0.250	0.47	0	1	1
Taurine	0.100	0.075	0.050	0.025	0.025	0.025	3.76	0	0	0
**NEAA**										
Ala	0.100	0.075	0.050	0.025	0.025	0.025	1.26	0	0.05	0.1
Asn	0.100	0.075	0.050	0.025	0.025	0.025	0.09	0	0.05	0.1
Asp	0.100	0.075	0.050	0.025	0.025	0.025	0.43	0	0.05	0.1
Glu	0.100	0.075	0.050	0.025	0.025	***0.050***	1.69	0	0.05	0.1
Gly	2.100	1.575	1.050	0.525	0.525	0.525	1.38	0	0.05	0.1
Pro	0.100	0.075	0.050	0.025	0.025	0.025		0	0.05	0.1
Ser	0.100	0.075	0.050	0.025	0.025	0.025	0.26	0	0.05	0.1
**EAA**										
Arg	0.300	0.225	0.150	0.075	0.075	***0.150***	0.03	0	0.3	0.6
Cys	0.050	0.038	0.025	0.013	0.013	***0.025***		0	0.05	0.1
His	0.100	0.075	0.050	0.025	0.025	***0.050***	0.07	0	0.1	0.2
Iso	0.200	0.150	0.100	0.050	0.050	***0.100***	0.12	0	0.2	0.4
Leu	0.200	0.150	0.100	0.050	0.050	***0.100***	0.22	0	0.2	0.4
Lys	0.200	0.150	0.100	0.050	0.050	***0.100***	0.24	0	0.2	0.4
Met	0.050	0.038	0.025	0.013	0.013	***0.025***	0.08	0	0.05	0.1
Phe	0.100	0.075	0.050	0.025	0.025	***0.050***	0.12	0	0.1	0.2
Thr	0.200	0.150	0.100	0.050	0.050	***0.100***	0.33	0	0.2	0.4
Trp	0.025	0.019	0.013	0.006	0.006	***0.013***	0.04	0	0.025	0.05
Tyr	0.100	0.075	0.050	0.025	0.025	***0.050***	0.14	0	0.1	0.2
Val	0.200	0.150	0.100	0.050	0.050	***0.100***	0.21	0	0.2	0.4
Total =	**15.21**	**11.41**	**7.61**	**3.80**	**5.33**	**4.26**	**21.20**	**30.89**	**13.48**	**14.78**
(mmol nutrients/L)										
Reference							9	46	13	12

Although the concentrations of nutrients present in most embryo culture media bear some similarity to the compositions of oviductal and uterine fluids (Tables 1 and 2), recent metabolomic studies suggest murine, bovine, and human embryos utilize less than 20% of the metabolites available to them in culture medium^17^. It is perhaps not surprising that embryos can develop equally well in a wide variety of media given the amounts of nutrients present in these media far exceed the amounts used by the embryo. In the current study, we hypothesized that viable embryos could develop in media with significantly reduced nutrient concentrations. Media with reduced nutrient concentrations were then supplemented with specific nutrients at specific stages of culture, providing a novel means of formulating embryo culture media.

## Materials and Methods

Unless specified otherwise, all chemicals were supplied from Millipore Sigma (St Louis, MO, USA. Nonessential amino acids (NEAA), essential amino acids (EAA), vitamins, insulin, transferrin, and selenium (ITS), and gentamicin were purchased from Corning (Mediatech, Manassas, VA, USA). All laboratory supplies and chemical reagents were screened for their ability to support embryo development using a sensitive mouse embryo assay before use in experiments^18^.

### Animals

Outbred (Hsd:NSA CF1) female mice (Envigo, Indianapolis, IN) 4 to 8 weeks of age were used for this study. Animals were maintained on a 14 h:10 h light:dark cycle with ad libitum access to food and water for the duration of the study. All animal experiments were approved by the Fertility Laboratories of Colorado Ethics in Research Committee and conducted in accordance with guidelines outlined in the U.S. National Research Council’s Guide for the Care and Use of Laboratory Animals^19^.

### Oocyte collection, *in vitro* fertilization and embryo culture

Embryos were produced from *in vivo* matured oocytes and IVF to best approximate the procedures used in human ART. Female mice were treated with 5 IU pregnant mare serum gonadotropin (PMSG; Calbiochem® EMD Millipore, Billerica, MA, USA), followed by 5 IU human chorionic gonadotropin (hCG; Calbiochem® EMD Millipore) 48 h later. Spermatozoa were collected from B6D2F1 males (3 to 4 months of age; Envigo) and capacitated for one hour in mouse oocyte fertilization medium (mOFM)^20^. Females were euthanized 16 h post-hCG and oviducts were collected in MOPS-buffered collection medium with 5% fetal calf serum (FCS; HyClone Laboratories, Logan, UT, USA). The cumulus-oocyte masses were placed into 50 µL drops of mOFM containing capacitated sperm (1 ×10^6^ sperm/mL), and gametes were co-incubated for 6 h. After IVF, zygotes with visible pronuclei were randomly assigned to treatments and placed into culture (10 ± 2 embryos per 20 µL drop) at 37 °C in 7.5% CO_2_ and 6.5% O_2_ (equivalent to 6.0% CO_2_ and 5.0% O_2_ at sea level) in step one Optimized Embryo Culture (OEC1) medium (Table 1)^21^. After 48 h in culture, embryos were transferred to step two OEC (OEC2, Table 2) and cultured in the same conditions. Blastocyst development was evaluated at 96 h (day 4) and blastocyst hatching at 112 h (days 4 and 5) of culture.

### Blastocyst cell number and allocation

For quantification of inner cell mass (ICM) and trophectoderm (TE) cells, day 5 hatching or fully hatched blastocysts were fixed in 4% paraformaldehyde (Electron Microscopy Sciences, Hatfield, PA, USA). Antibodies against SRY (sex determining region Y)-box 2 (SOX2; Biogenex, Fremont, CA, USA, rabbit monoclonal) and Caudal-Type Homeobox Protein 2 (CDX2; Biogenex, mouse monoclonal) were used to identify ICM and TE cells, respectively^22,23^. Secondary antibodies Alexa Fluor 488 donkey anti-rabbit IgG and Alexa Fluor 555 goat anti-mouse IgG (Invitrogen, Thermo Fisher Scientific) were used for SOX2 and CDX2, respectively. Blastocysts were mounted using ProLong Gold Antifade Mountant with DAPI (Life Technologies, Carlsbad, CA, USA) and evaluated (400×) using a fluorescent microscope and MetaMorph software (Molecular Devices, Sunnyvale, CA, USA). Total cell number was calculated as the number of SOX2 positive cells plus the number of CDX2 positive cells.

### ATP concentration

The concentration of ATP was analyzed in individual hatching and fully hatched blastocysts collected on day 5 of culture. Blastocysts were washed and collected in 10 µL phosphate-buffered saline (PBS) with 0.01% polyvinylpyrrolidone (PVP), snap frozen in liquid nitrogen, and stored at −80 °C until analysis. ATP concentrations were determined using the ATP Bioluminescent Somatic Cell Assay Kit (Sigma), as described previously with minor modifications^21,24^. Sample and standard mixtures were transferred to reaction wells in an opaque 96 well plate, and the amount of light emitted was measured using a Synergy 2 plate reader for luminescence (BioTek, Winooski, VT). Background luminescence was subtracted from all readings. ATP concentration was calculated by comparison against a standard curve.

### Embryo kinetics

Embryos were cultured individually in EmbryoSlides® in the EmbryoScope® (Vitrolife) time lapse system. Dishes were prepared with 25 µL per well of control or treatment medium and covered with mineral oil. Embryos were moved to the second step medium after 48 h of culture. Throughout culture, an image was obtained of each embryo in each well approximately every 10 min in order to create a composite video of kinetic progression. Key developmental time points (cell divisions, morula compaction, blastocyst cavitation, blastocyst hatching) were annotated using the EmbryoViewer software.

### Embryo metabolomics

To assess metabolic activity of individual embryos, 10 µL of spent (embryo present) and unspent (no embryo present) media was collected from each EmbryoSlide® well. Media was collected to ensure no oil contamination, placed into a microcentrifuge tube and snap-frozen in liquid nitrogen. Samples were stored at −80 °C until analysis at Colorado State University Proteomics and Metabolomics Core. Concentrations of nutrients were determined using gas chromatography (GC) and mass spectrometry (MS) with relative quantification as previously described^17^. Aliquots (1 µl) of each sample were injected in a 1:10 split ratio twice in discrete randomized blocks (n = 2 injections/sample) for analysis using a Trace GC Ultra coupled to a Thermo ISQ mass spectrometer (Thermo Scientific). Separation occurred using a 30-m TG-5MS column (0.25 mm i.d. and 0.25 µm film thickness; Thermo Scientific). A matrix of molecular features defined by retention time and mass (*m/z*) was generated using XCMS software in R for feature detection and alignment^25^. Raw peak values were normalized against total ion signal in R, outliers were detected based on total signal and principal component 1 of principle component analysis, and the mean area of the chromatographic peak was calculated among replicate injections (n = 2). Features were grouped with Automated Mass Spectral Deconvolution and Identification System^26^ and metabolites were annotated by matching retention time and mass spectra to NIST v11 and v12, Golm, Metlin, and Massbank metabolite databases. Initial experiments indicated that glutamine in the samples was converted to pyroglutamate during sample preparation and/or analysis^17^. Therefore, pyroglutamate concentrations are considered to represent the concentration of glutamine in the samples.

### Gene expression analysis

Expression of genes involved in fatty acid metabolism [acyl-Coenzyme A dehydrogenase, long-chain (*Acadl*), acyl-CoA synthetase long-chain family member 3 (*Acsl3*), carnitine palmitoyltransferase 1b (*Cpt1b*), carnitine palmitoyltransferase 2 (*Cpt2*)] and carbohydrate metabolism [hexokinase 1 (*Hk1*), lactate dehydrogenase A (*Ldha*), mitochondrially encoded cytochrome c oxidase II (*mt-Co2*), pyruvate dehydrogenase E1 alpha 1 (*Pdha1*), and pyruvate dehydrogenase kinase 1 (*Pdk1*)] were analyzed (Supplementary Table 1). Data were normalized using expression of the housekeeping gene peptidylprolylisomerase A (*Ppia*). A total of 5 biological replicates (pools of 6 hatching or fully hatched blastocysts on day 5) for each treatment were used for quantitative real time PCR (q-PCR). RNA was isolated using PicoPure RNA Isolation Kit (Thermofisher Scientific, Waltham, MA) and subjected to cDNA synthesis using qScript™ cDNA Supermix (Quanta Biosciences, Gaithersburg, MD) following the manufacture’s protocol. After 1:4 dilutions, 1.5 µL cDNA was used for q-PCR analysis. A total 10 µL PCR reaction was performed in duplicate using 5 µL Power SYBR™ Green PCR Master Mix (Applied Biosystems, Foster City, CA), 1 µL of 10 µM primer mix (i.e. forward and reverse), 1.5 µL of diluted cDNA sample, and 2.5 µL RNase free water. The qPCR program was: 50 °C for 2 min for first cycle, 95 °C for 10 min for second cycle followed by 40 cycles of amplification step at 95 °C for 10 s and 60 °C for 1 min. A melting curve was analyzed for each experiment to assess the specificity of primer amplification. Relative gene expression was calculated using the 2 − ΔΔCt method^27^.

### Embryo outgrowth

On day 5 of culture, hatching and fully hatched blastocysts that had been cultured in control medium or reduced nutrient treatment medium were transferred to fibronectin-coated 24-well plates (Corning, NY, USA) containing 500 µL control medium, one embryo per well, in order to assess embryo viability^28^. Embryo attachment to the surface of the dish was determined after 60 h. After 108 h, a digital image of the entire embryo was taken and used to determine the total area (arbitrary units) of embryo outgrowth using MetaMorph software.

### Embryo Transfer

Embryos were cultured in groups as previously described and expanded and hatching blastocysts were selected for surgical embryo transfer on day 3.5 (~84 h) of culture. Swiss Webster females (3–6 months of age; Harlan Laboratories, Indianapolis, IN) determined to be in estrus or proestrus^29^ were paired with vasectomized CF1 males (3–6 months of age; Harlan Laboratories, Indianapolis, IN) for mating. Those females confirmed to be pseudopregnancy by the presence of a vaginal plug were used as embryo recipients. Six to 8 embryos from the control and treatment groups were surgically transferred into separate horns of the same female on day 2.5 of pseudpopregnancy. Implantation and fetal development was determined on day 14.5 of development. Fetal crown-rump length, fetal weight, and placental diameter and weight were assessed for each fetus.

### Statistical analysis

To evaluate embryo development, each embryo was scored as a 1 or 0 depending on whether or not it had cleaved (24 h post-insemination, hpi), formed a blastocyst (day 4) or initiated hatching (day 5). Data was analyzed using two-way ANOVA, with treatment as a fixed factor and replicate as a random factor. If significant, the Bonferonni multiple comparison test was used to determine differences between treatments.

A one-way ANOVA with Bonferonni multiple comparison test was used to analyze cell number, ATP concentration, outgrowth attachment and area, and embryo kinetics. Gene expression was analyzed using one-way ANOVA with a Tukey’s test for multiple comparisons. Embryo transfer data was analyzed using Chi-square for pregnancy and implantation data, or GLM ANOVA for fetal and placental descriptors, nested by female. In all analyses, p < 0.05 was considered a significant difference. All means are presented ± SEM.

To assess blastocyst metabolomic data, a one-way ANOVA with Bonferroni multiple comparison test was used to analyze metabolite concentrations. Significant increases or decreases in the concentration of a metabolite in a medium sample that contained an embryo compared to medium samples that did not contain an embryo are indicative of production or consumption, respectively, of the metabolite by the embryo within each treatment^30^. The change in concentration of nutrients in the medium following embryo culture was expressed as the percent change during the culture period and used for comparisons between treatments.

### Experimental design

#### Reduction of nutrients in culture medium

To determine the effects of reducing nutrient concentrations in culture medium on developmental competence and quality of resulting embryos, culture media were prepared with 75% (OEC75), 50% (OEC50), and 25% (OEC25) of the concentrations of nutrients (carbohydrates, amino acids, vitamins, and EDTA) present in our standard (100%), control medium (OEC100; Tables 1 and 2)^21^. All other medium components (salts, bicarbonate, albumin, etc.) were present at concentrations found in the control medium to maintain consistent pH and osmolality, which was confirmed in control and treatment media. Fresh media was prepared for each replicate. Trace amounts of fatty acids associated with the recombinant human albumin, as well as endogenous pools of fatty acids in the embryos^31^, are possible energy sources that were available in equal quantities to embryos in all treatments. Effects of these treatments on embryo development, blastocyst cell number and allocation, blastocyst ATP content, developmental kinetics, and gene expression were examined (Supplementary Table 2, Exp. 1).

#### Developmental Rescue by Glucose, EDTA, Alanyl-glutamine, or Pyruvate and Lactate

Based upon results of the first experiment, our next objective was to determine if specific nutrient(s) were able to restore development of embryos cultured in OEC25 (Supplementary Table 2, Exp. 2), which will be referred to as reduced nutrient (RN) medium from this point forward. Glucose, EDTA, alanyl-glutamine, and pyruvate and L-lactate (PL) were individually added to RN such that final concentrations of these nutrients were equivalent to those found in OEC50 (Tables 1 and 2). EDTA is only present during the step one of culture, and as such was only replaced in step one. Pyruvate and lactate were studied together to maintain a consistent pyruvate:lactate ratio.

#### Stage-specific effects of Pyruvate and Lactate

To determine if the effects of pyruvate and lactate (PL) on embryo development in RN media were stage-specific, PL at concentrations found in OEC50 (Tables 1 and 2) were further evaluated in either the first (RN1 + PL: 0.15 mM P and 5.0 mM L), second (RN2 + PL: 0.05 mM P and 3.0 mM L), or both steps of culture (Supplementary Table 2, Exp. 3). Effects of these treatments on embryo development and blastocyst cell number and allocation were examined. Embryos were also cultured in the control medium (OEC100) or RN + PL in both steps of culture (RN1 + PL/RN2 + PL) and embryo development, blastocyst cell number and allocation, ATP content, developmental kinetics, and metabolism were examined (Supplementary Table 2, Exp. 4).

#### Effects of nonessential amino acids in RN medium

We next focused on the impact of adding non-essential amino acids (NEAA) at OEC50 concentrations to RN1 in the presence (OEC50 concentrations, Tables 1 and 2) or absence of PL (RN1, RN1 + PL, RN1 + NEAA, RN1 + PL + NEAA) (Supplementary Table 2, Exp. 5A). All embryos were cultured in RN2 + PL. In the next experiment, embryos were cultured in RN1 + PL, and NEAA and/or PL (OEC50 concentrations) were added to RN2 (RN2, RN2 + PL, RN2 + NEAA, RN2 + PL + NEAA). Embryo development and blastocyst cell number and allocation were examined in both experiments (Supplementary Table 2, Exp. 5B).

#### Effects of essential amino acids or individual amino acids in RN + PL medium

The effects of essential amino acids (EAA), as well as the individual effects of arginine (Arg)^32^, proline (Pro)^33^, and glutamate (Glu)^34^, were evaluated in RN2 at 50% of the control concentrations following culture in RN1 + PL (Supplementary Table 2, Exp. 6). Effects of these treatments on embryo development and blastocyst cell number and allocation were examined.

Finally, embryos were cultured in control OEC100 medium or the optimized reduced nutrient medium (RN1 + PL/RN2 + EAA + GLU). Effects of these treatments on embryo development, blastocyst cell number and allocation, ATP content, gene expression, and implantation and fetal development following surgical embryo transfer were examined (Supplementary Table 2, Exp. 7).

## Results

### Reduction of nutrients in culture medium

**Figure 1:**
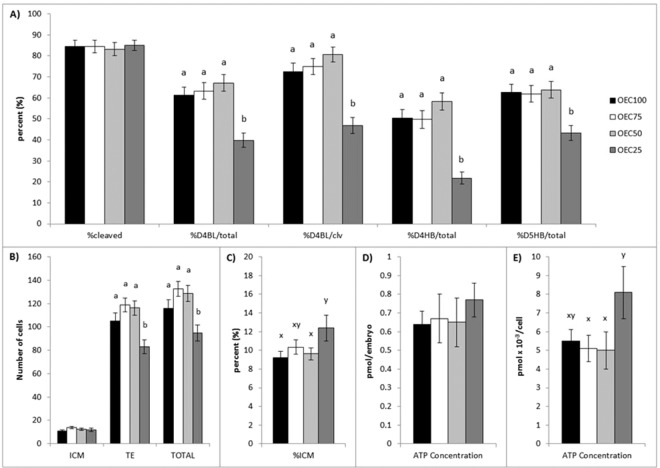
Effect of reducing culture medium nutrient concentrations on preimplantation mouse embryo development, cell number, and ATP concentration. Embryos were cultured in medium with control nutrient concentrations (OEC100; black bars), and reduced nutrient concentrations of 75% (OEC75; white bars), 50% (OEC50; light gray bars) and 25% (OEC25; dark gray bars). (**A**) The proportion of presumptive zygotes that cleaved to the two cell stage (% cleaved), and developed to the blastocyst (BL) and hatching blastocyst (HB) stage on day 4 (D4) and day 5 (D5) of culture were assessed. Values are expressed as a percentage of total zygotes cultured (total) and of cleaved embryos (clv). (**B**) Average number of inner cell mass (ICM; SOX2 positive), trophectoderm (TE; CDX2 positive), and total cells, and (**C**) percentage of ICM cells in individual hatching and fully hatched blastocysts. Average ATP concentration of individual blastocysts expressed (**D**) per embryo and (**E**) per cell. Data are presented as the mean ± s.e.m. Different superscripts indicate significant differences (^ab^p ≤ 0.05) or trends (^xy^p < 0.09) between treatments within a given endpoint.

Reducing nutrient concentrations had no effect on embryo cleavage to the 2-cell stage. Only when embryos were cultured with 25% nutrient concentrations, equivalent to a 75% reduction in available nutrients, was development to the blastocyst stage on day 4 decreased (p < 0.01) compared to media with 100% (control), 75%, or 50% nutrients, expressed as a percentage of both total zygotes cultured (39.8 ± 3.4%, 67.1 ± 3.9%, 63.3 ± 4.0%, 61.2 ± 4.0%, respectively) and of cleaved embryos (46.9 ± 3.8%, 80.6 ± 3.6%, 75.0 ± 3.9%, 72.6 ± 4.0%, respectively; Fig. 1A). Additionally, the incidence of blastocyst hatching was lower (p < 0.01) in OEC25 compared to OEC50, OEC75, and OEC100 on day 4 (21.8 ± 2.9%, 58.4 ± 4.1%, 49.7 ± 4.1%, 50.3 ± 4.1% per zygote, respectively) and day 5 (43.2 ± 3.5%, 63.8 ± 4.0%, 61.9 ± 4.0%, 62.6 ± 4.0% per zygote, respectively) of culture.

Cell number and allocation in day 5 hatching and fully hatched blastocysts that developed in OEC25 also differed from those cultured in OEC50, OEC75, and OEC100, with fewer (p < 0.01) TE (83.0 ± 5.9, 116.2 ± 6.1, 119.1 ± 5.9, 105.1 ± 6.9, respectively) and total (94.8 ± 6.7, 128.6 ± 6.8, 132.8 ± 6.4, 115.8 ± 7.5, respectively) cells (Fig. 1B). There was no difference between treatments in the number of ICM cells (OEC25, 11.8 ± 1.5; OEC50, 12.4 ± 1.0; OEC75, 13.7 ± 1.1; OEC100, 10.7 ± 1.1). Blastocysts cultured in OEC25 tended (p = 0.08) to have a higher percentage of ICM cells than those in OEC100 and OEC50 (12.40 ± 1.4%, 9.23 ± 0.7%, 9.63 ± 0.7%, respectively; Fig. 1C).

There were no differences in ATP concentration between day 5 hatching blastocysts cultured in OEC100 (0.64 ± 0.07 pmol per embryo), OEC75 (0.67 ± 0.13 pmol per embryo), OEC50 (0.65 ± 0.13 pmol per embryo), or OEC25 (0.77 ± 0.09 pmol per embryo; Fig. 1D). However, taking into account the average total cell number for each treatment, the amount of ATP per cell tended to be greater in OEC25 compared to OEC75 and OEC50 (8.1 ± 1.4 ×10^−3^ pmol per cell, 5.1 ± 0.7 ×10^−3^ pmol per cell, 5.0 ± 1.0 ×10^−3^ pmol per cell, respectively), but was not different than embryos from OEC100 (5.5 ± 0.6 ×10^−3^ pmol per cell; Fig. 1E).

Embryos cultured in OEC25 took longer (p ≤ 0.01) than embryos grown in OEC100 to develop into an 8-cell embryo (58.7 ± 1.3, 55.1 ± 1.1 hpi, respectively), form a morula (73.1 ± 1.2, 67.0 ± 1.5 hpi, respectively), initiate blastocoel formation (96.0 ± 1.8, 85.6 ± 1.5 hpi, respectively), reach the blastocyst stage (101.6 ± 2.1, 89.5 ± 1.5 hpi, respectively), and initiate hatching (105.8 ± 2.2, 94.8 ± 1.8 hpi, respectively). Additionally, time to compaction tended (p = 0.06) to be longer in OEC25 (87.4 ± 2.1 hpi) than OEC100 (79.4 ± 2.2 hpi; Supplementary Fig. 1).

**Figure 2:**
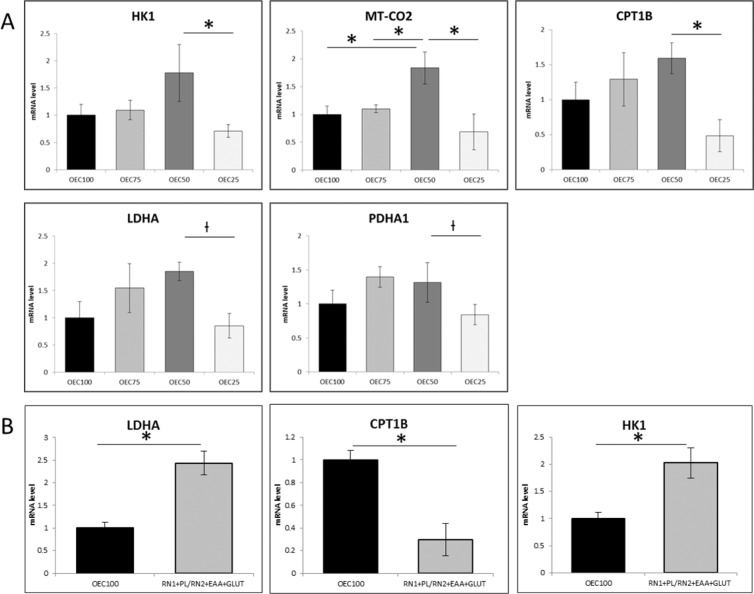
Relative gene expression in day 5 blastocysts cultured in (**A**) control (OEC100) or reduced nutrient (OEC75, OEC50, OEC25) culture medium, and (**B**) control (OEC100) or optimized reduced nutrient (RN1 + PL/RN2 + EAA + GLU) culture medium. *Cpt1b*, carnitine palmitoyltransferase 1b; *Hk1*, hexokinase 1; *Ldha*, lactate dehydrogenase A; *mt-Co2*, mitochondrially encoded cytochrome c oxidase II; *Pdha1*, pyruvate dehydrogenase E1 alpha 1. Superscripts indicate significant differences (*p ≤ 0.01) or trends (†p < 0.07) between treatments within a given gene.

The expression of genes related to metabolism was analyzed in embryos cultured in OEC100, OEC75, OEC50, and OEC25. Relative mRNA levels were based on a control OEC100 level of 1.0. Elevated expression of *Cpt1b* and *Hk1* was observed in OEC50 compared to OEC25. Expression of *mt-Co2* was higher in OEC50 compared to OEC25, OEC75, and OEC100. There was a tendency for higher expression of *Ldha* (p = 0.05) and *Pdha1* (p = 0.07) in OEC50 compared to OEC25 (Fig. 2A). No differences were found in the expression of *Acadl*, *Acsl3*, *Cpt2*, or *Pdk1* between treatments.

### Developmental rescue by glucose, EDTA, alanyl-glutamine, or Pyruvate and Lactate

**Figure 3:**
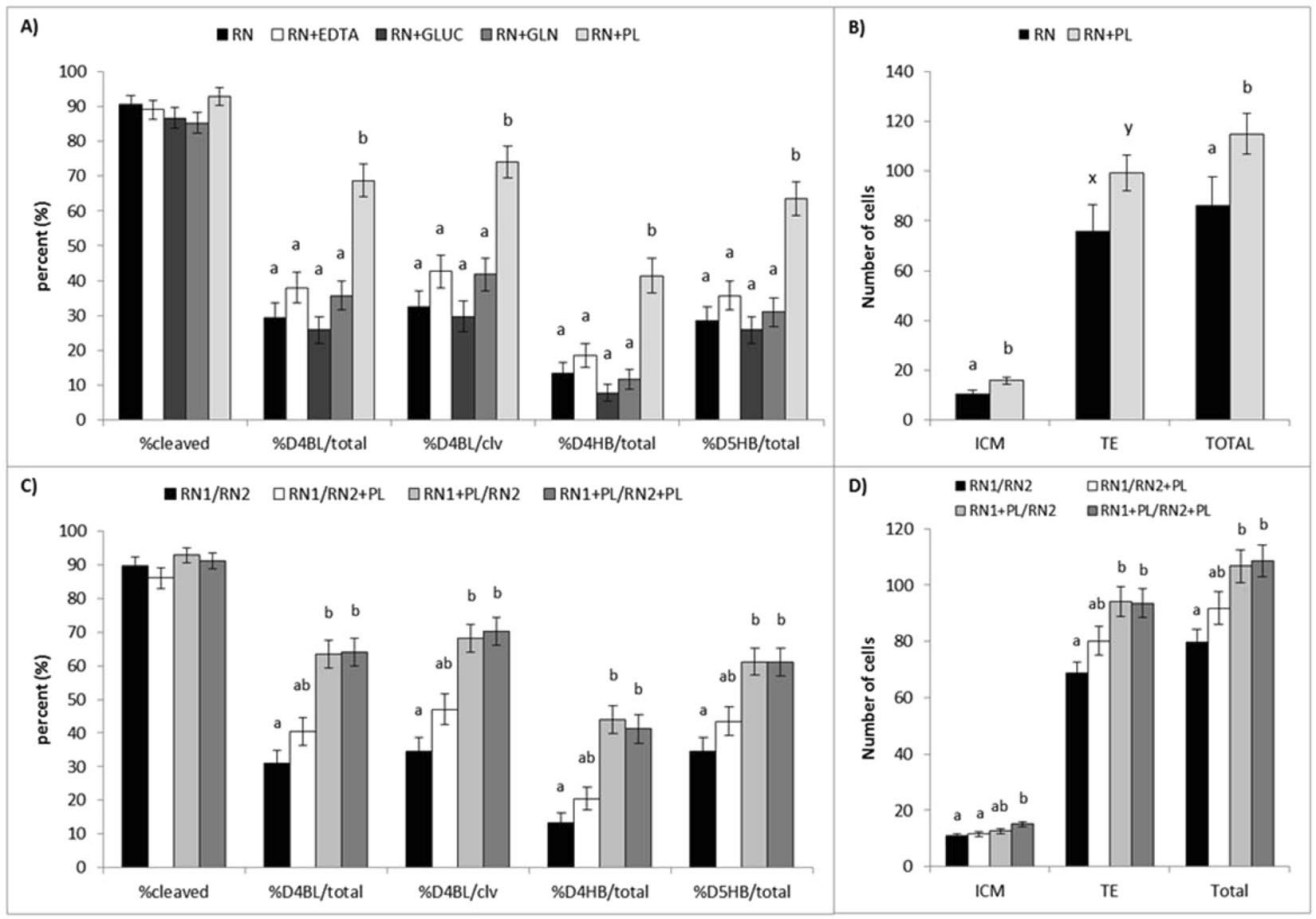
Addition of individual nutrients to reduced nutrient media (OEC25; RN) in an attempt to rescue embryo development and cell number. Embryos were cultured in reduced nutrient medium (RN; black bars), and RN supplemented with EDTA (white bars), glucose (GLUC; dark gray bars), alanyl-glutamine (GLN; medium gray bars), or pyruvate and L-lactate (PL; light gray bars) at 50% concentration in both steps of culture. (**A**) The proportion of presumptive zygotes that cleaved to the two cell stage (% cleaved), and developed to the blastocyst (BL) and hatching blastocyst (HB) stage on day 4 (D4) and day 5 (D5) of culture were assessed. Values are expressed as a percentage of total zygotes cultured (total) and of cleaved embryos (clv). (**B**) The number of inner cell mass (ICM; SOX2 positive), trophectoderm (TE; CDX2 positive), and total cells in hatching and fully hatched blastocysts. (**C**) Embryo development and (**D**) average number of ICM, TE, and total cells of individual embryos cultured in reduced nutrient (RN) culture medium with step-wise addition of pyruvate and lactate (PL). Data are presented as the mean ± s.e.m. Different superscripts indicate significant differences (^ab^p ≤ 0.02) or trends (^xy^p = 0.07) between treatments within a given endpoint.

When glucose, EDTA, alanyl-glutamine, or pyruvate and L-lactate (PL), were added to RN (OEC25) at the concentrations present in OEC50, only RN + PL significantly increased (p < 0.01) day 4 blastocyst development compared to RN, both per total zygotes (68.7 ± 4.7%, 29.4 ± 4.1%, respectively) and per cleaved embryos (74.0 ± 4.6%, 32.5 ± 4.4%, respectively; Fig. 3A). Culture in RN + PL also significantly increased (p < 0.01) blastocyst hatching compared to RN on day 4 (41.4 ± 5.0%, 13.5 ± 3.1% per zygote, respectively) and day 5 (63.6 ± 4.9%, 28.6 ± 4.0% per zygote, respectively) of culture. Glucose, EDTA, and alanyl-glutamine had no effect on any analyzed embryo developmental parameter compared to RN.

Culture of embryos in RN + PL significantly increased total cell number in hatching blastocysts compared to RN (115.0 ± 8.2, 86.5 ± 11.3, respectively), as well as the number of ICM cells (15.8 ± 1.4, 10.5 ± 1.4; Fig. 3B). Blastocysts cultured in RN + PL tended (p = 0.07) to have an increased number of TE cells compared to RN (99.2 ± 7.1 vs 76.0 ± 10.5, respectively). There was no significant difference in the percentage of ICM cells of embryos cultured in RN (12.1 ± 1.9%) and RN + PL (13.8 ± 1.0%).

### Stage-specific effects of Pyruvate and Lactate

The addition of PL during the first (RN1 + PL/RN2), second (RN1/RN2 + PL) or both (RN1 + PL/RN2 + PL) steps of culture did not affect embryonic cleavage compared to RN medium alone (RN1/RN2). Development to the blastocyst stage on day 4 was significantly lower (p < 0.01) in RN1/RN2 compared to RN1 + PL/RN2 and RN1 + PL/RN2 + PL as a percentage of total zygotes (30.9 ± 4.0%, 63.3 ± 4.1%, 64.0 ± 4.1%, respectively; Fig. 3C) and of cleaved embryos (34.4 ± 4.3%, 68.2 ± 4.1%, 70.2 ± 4.1%, respectively). In addition, blastocyst hatching (per total zygotes) was significantly lower (p < 0.02) in RN1/RN2 than RN1 + PL/RN2 and RN1 + PL/RN2 + PL on both day 4 (13.2 ± 2.9%, 43.9 ± 4.2%, 41.2 ± 4.2%, respectively) and day 5 (34.6 ± 4.1%, 61.2 ± 4.1%, 61.0 ± 4.2%, respectively). There was no additional benefit of adding PL to RN2 following culture in either RN1 or RN1 + PL on blastocyst development or hatching compared to RN1/RN2 or RN1 + PL/RN2, respectively (Fig. 3C).

Embryos cultured in RN1/RN2 and RN1/RN2 + PL had fewer (p < 0.01) ICM cells than those in RN1 + PL/RN2 + PL (11.0 ± 0.7, 11.6 ± 1.0, 15.1 ± 0.9, respectively; Fig. 3D). There were also fewer (p < 0.01) TE cells in blastocysts cultured in RN1/RN2 than those in RN1 + PL/RN2 and RN1 + PL/RN2 + PL (68.8 ± 3.9, 94.0 ± 5.3, 93.5 ± 5.1, respectively). Additionally, RN1/RN2 differed (p < 0.01) from RN1 + PL/RN2 and RN1 + PL/RN2 + PL in total cell number (79.8 ± 4.4, 106.7 ± 5.8, 108.6 ± 5.7, respectively; Fig. 3D). There were no significant differences in the percentage of ICM cells between treatments (RN1/RN2, 14.2 ± 0.8%; RN1 + PL/RN2, 12.1 ± 0.7%; RN1/RN2 + PL, 12.7 ± 1.0%; RN1 + PL/RN2 + PL, 14.5 ± 0.7%).

**Figure 4:**
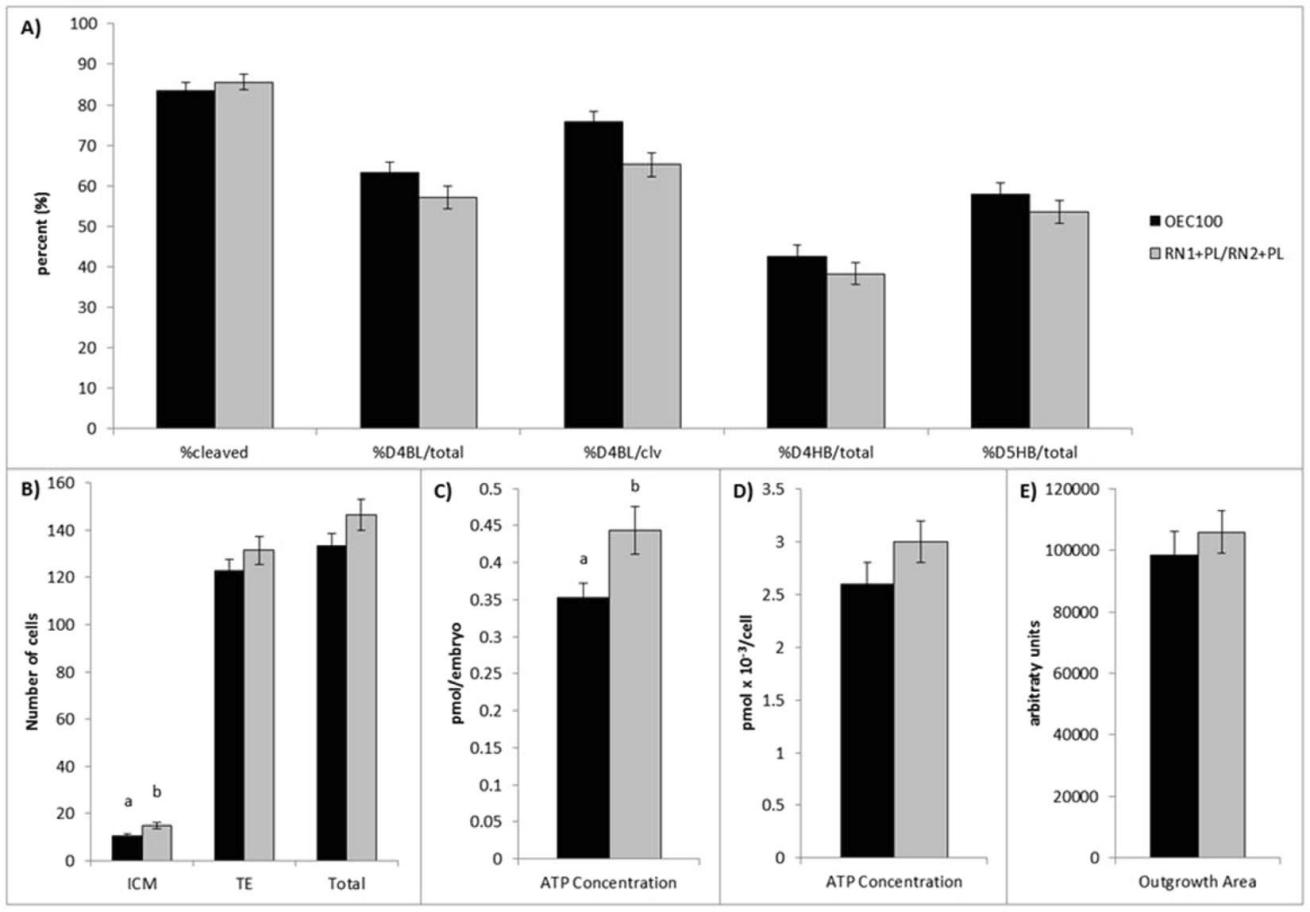
Comparison of control (OEC100; black bars) and reduced nutrient culture medium (RN1 + PL/RN2 + PL; gray bars) for their ability to support blastocyst development and embryo quality in the mouse. (**A**) The proportion of presumptive zygotes that cleaved to the two cell stage (% cleaved), and developed to the blastocyst (BL) and hatching blastocyst (HB) stage on day 4 (D4) and day 5 (D5) of culture were assessed. Values are expressed as a percentage of total zygotes cultured (total) and of cleaved embryos (clv). (**B**) The number of inner cell mass (ICM; SOX2 positive), trophectoderm (TE; CDX2 positive), and total cells in hatching and fully hatched blastocysts. Average ATP concentration of individual blastocysts expressed (**C**) per embryo and (**D**) per cell, and (**E**) outgrowth area of hatching and hatched blastocysts cultured on fibronectin-coated plates. Data are presented as the mean ± s.e.m. Different superscripts indicate significant differences (p ≤ 0.05) between treatments within a given endpoint.

Comparing RN1 + PL/RN2 + PL to our standard control medium (OEC100), we observed no differences in embryo cleavage, and no differences in blastocyst development on day 4 of culture per total zygote (57.1 ± 2.8% vs. 63.2 ± 2.7%, respectively) or per cleaved embryo (65.2 ± 2.9% vs. 75.7 ± 2.7%, respectively). Similarly, there were no differences in blastocyst hatching between OEC100 and RN1 + PL/RN2 + PL on day 4 (42.5 ± 2.8% vs 38.2 ± 2.7%, respectively) or day 5 (57.8 ± 2.8% vs 53.6 ± 2.8%, respectively) of culture (Fig. 4A).

Culture in RN1 + PL/RN2 + PL increased (p < 0.01) the number of ICM cells in blastocysts compared to culture in OEC100 (15.0 ± 1.3 vs 10.5 ± 0.9, respectively; Fig. 4B). Additionally, RN1 + PL/RN2 + PL blastocysts had a significantly higher percentage of ICM cells than OEC100 (10.2 ± 0.8% vs 7.8 ± 0.5%). There was no significant difference observed in TE (123.0 ± 4.7, 131.5 ± 6.0) or total (133.5 ± 5.2, 146.4 ± 6.6) cell numbers between embryos cultured in OEC100 and RN1 + PL/RN2 + PL.

The concentration of ATP in RN1 + PL/RN2 + PL treated blastocysts was higher (p ≤ 0.02) than those cultured in OEC100 (0.44 ± 0.03 pmol per embryo, 0.35 ± 0.02 pmol per embryo, respectively; Fig. 4C). However, when normalized to ATP per cell using the average total cell number for each treatment, there was no difference (OEC100, 2.6 ± 0.2 pmol x 10^−3^ per cell; RN1 + PL/RN2 + PL, 3.0 ± 0.2 pmol x 10^−3^ per cell; Fig. 4D). Outgrowth attachment was not different between OEC100 and RN1 + PL/RN2 + PL at 60 h (52.5 ± 8.0%, 50.0 ± 7.8%, respectively) and 108 h (78.9 ± 6.7%, 71.4 ± 7.1%, respectively). Outgrowth area after 108 h was not different between embryos cultured in OEC100 (98,371.4 ± 7768.6 arbitrary units) and RN1 + PL/RN2 + PL (105,939.6 ± 6861.3 arbitrary units; Fig. 4E).

Embryos cultured in RN1 + PL/RN2 + PL tended (p = 0.09) to be slower than OEC100 to reach the 6-cell (55.1 ± 0.7, 53.6 ± 0.5 hpi, respectively) and 8-cell (57.0 ± 0.7, 55.2 ± 0.7 hpi, respectively) stages. Time to compaction was also longer (p = 0.02) in RN1 + PL/RN2 + PL (82.3 ± 1.2 hpi) than OEC100 (78.5 ± 1.1 hpi; Supplementary Fig. 2).

**Figure 5:**
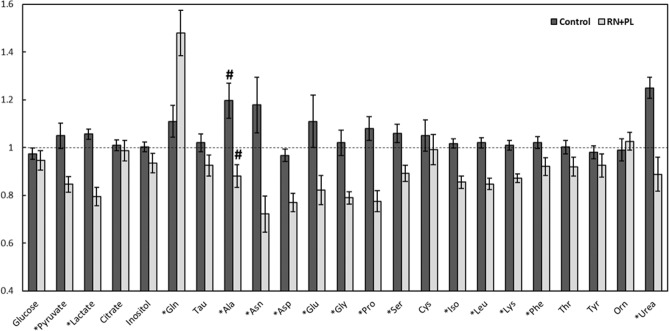
Relative change in the concentrations of nutrients present in control or reduced nutrient medium (RN) with pyruvate and lactate (PL) added at 50% of the control concentrations following the culture of an individual embryo in 25 µl of medium for 60 h (48 to 108 h). The amount of each nutrient is shown as a proportion of what was present in a 25 µl drop of the same medium that did not contain an embryo (dashed line). *Indicates a significant difference (P < 0.05) in the relative consumption or production of that nutrient between embryos in the two media. #Indicates significant (P < 0.05) production of Ala by embryos in the control medium and significant (P < 0.05) consumption of Ala by embryos in the RN + PL medium compared to samples of the same media that were cultured without an embryo.

Embryos cultured in RN1 + PL/RN2 + PL consumed (p < 0.04) alanine (−13.7%), glycine (−23.2%), leucine (−16.5%), and lysine (−14.6%), and tended (p = 0.09) to consume isoleucine (−15.9%) in the second step of culture. Embryos cultured in OEC100 did not consume significant quantities of any nutrient, but did produce alanine (+22.5%), and tended (p = 0.06) to produce asparagine (+21.3%) and proline (+10.7%). The relative change in nutrient concentrations used or produced in the second step of culture was different (p < 0.05) between OEC100 and RN1 + PL/RN2 + PL for alanine (119.6 ± 7.3% vs 88.1 ± 4.7%), asparagine (117.8 ± 11.7% vs 72.2 ± 7.5%), aspartic acid (96.7 ± 2.7% vs 77.0 ± 3.9%), glutamate (111.4 ± 11.0% vs 82.2 ± 6.1%), glycine (102.9 ± 5.3% vs 79.0 ± 2.6%), isoleucine (101.8 ± 1.9% vs 85.5 ± 2.5%), lactate (105.6 ± 2.1% vs 79.5 ± 3.8%), leucine (102.0 ± 2.2% vs 84.8 ± 2.3%), lysine (100.9 ± 2.1% vs 87.2 ± 1.8%), phenylalanine (101.9 ± 2.5% vs 92.1 ± 3.7%), proline (108.0 ± 5.0% vs 77.5 ± 4.4%), glutamine (111.5 ± 6.6% vs 147.9 ± 9.5%), pyruvate (105.4 ± 5.3% vs 84.6 ± 3.3%), serine (105.9 ± 3.8% vs 89.2 ± 3.4%), and urea (124.6 ± 4.4% vs 88.8 ± 7.1%), respectively (Fig. 5).

### Effects of nonessential amino acids in RN medium

There were no significant differences in embryo cleavage when embryos were cultured in RN1, RN1 + PL, RN1 + NEAA, or RN1 + PL + NEAA in step one, followed by culture of embryos from all treatments in RN2 + PL in step two. As observed in previous experiments, PL supplementation significantly (p < 0.01) improved development to the blastocyst stage (per zygote) on day 4 (64.7 ± 4.1%) compared to RN1 (44.4 ± 4.3%), while RN1 + NEAA (50.4 ± 4.3%) and RN1 + PL + NEAA (58.4 ± 4.2%) were not different. The percentage of hatching blastocysts (per zygote) on day 4 was greater in RN1 + PL (45.6 ± 4.3%) than RN1 (18.5 ± 3.4%) and RN1 + NEAA (24.1 ± 3.7%), while RN1 + PL + NEAA (33.6 ± 4.0%), was not different from either. Blastocyst hatching on day 5 tended (p = 0.09) to be higher in RN1 + PL (66.9 ± 4.0%) than RN1 (49.6 ± 4.3%) and RN1 + NEAA (50.3 ± 4.3%); RN1 + PL + NEAA (56.9 ± 4.2%) did not differ from any other treatment. Addition of PL, either alone or with NEAA, increased (p < 0.03) trophectoderm (TE) cell number (RN1 + PL, 131.8 ± 5.4; RN1 + PL + NEAA, 130.5 ± 5.0) compared to RN1 (112.8 ± 7.8) and RN1 + NEAA (111.8 ± 6.5), as well as the total number of cells (150.9 ± 6.0, 147.8 ± 5.5, 130.6 ± 8.0, 128.7 ± 7.0, respectively). There were no significant differences in the number of ICM cells between treatments.

Addition of PL and/or NEAA to the second step of culture, following culture in RN1 + PL, did not significantly alter blastocyst development (per embryo moved into step 2 treatment) on day 4 (RN2, 80.2 ± 4.5%; RN2 + PL, 80.7 ± 4.4%; RN2 + NEAA, 78.1 ± 4.6%; RN2 + PL + NEAA, 83.1 ± 4.1%) or hatching on day 5 (RN2, 77.8 ± 4.6%; RN2 + PL, 78.3 ± 4.6%; RN2 + NEAA, 81.7 ± 4.3%; RN2 + PL + NEAA, 85.5 ± 3.9%). In embryos cultured in RN2, RN2 + PL, RN2 + NEAA, or RN2 + PL + NEAA, the number of ICM (18.8 ± 0.9, 16.4 ± 0.8, 17.6 ± 0.9, 17.3 ± 0.9, respectively), TE (115.3 ± 6.1, 113.8 ± 5.7, 128.4 ± 6.2, 115.7 ± 5.7, respectively), and total (134.1 ± 6.4, 130.2 ± 6.0, 146.0 ± 6.6, 133.0 ± 5.9, respectively) cells did not differ. The percentage of ICM cells tended (p = 0.09) to be reduced in RN2 + NEAA (12.3 ± 0.6%) compared to RN2 (14.6 ± 0.7%).

### Effects of essential amino acids or individual amino acids in RN + PL medium

Addition of the entire set of EAA, Arg, Pro, or Glu to the second step of culture (RN2) did not affect blastocyst development on day 4 or blastocyst hatching on day 5 (per embryo moved to step 2) (Supplementary Table 3). There tended (p = 0.09) to be more ICM cells in blastocysts cultured in RN2 + EAA compared to +Arg and +Pro. There were no differences in TE or total cell numbers, or the percentage of ICM cells, between any of the treatments (Supplementary Table 3).

Based on the results of all experiments, a version of RN medium was formulated to contain PL at 50% of the standard concentration in step one (RN1 + PL), and EAA and Glu at 50% of the standard concentration in step 2 (RN2 + EAA + Glu). We removed PL from RN2 based on experiments 3 and 5 (Supplementary Table 2, Fig. 3) that failed to demonstrate any beneficial effect of including additional PL in step 2 of culture. In addition, EAA and Glu were included in RN2 based on the small, non-significant increases in ICM, TE and total cell number and day 5 blastocyst hatching, respectively (Supplementary Table 3). There were no differences in cleavage between zygotes cultured in OEC100 or RN1 + PL/RN2 + EAA + Glu. Similarly, there were no differences in blastocyst development on day 4 per total zygote, as well as no differences in blastocyst hatching on day 5 (Supplementary Table 4). However, there was a tendency (p = 0.09) for more hatching blastocysts per total zygotes on day 4 in RN1 + PL/RN2 + EAA + Glu compared to OEC100 (Supplementary Table 4).

Compared to those cultured in OEC100, blastocysts cultured in RN1 + PL/RN2 + EAA + Glu had significantly (p < 0.01) more TE and total cells (Supplementary Table 5). There was no difference in the number of ICM cells or percentage of ICM cells (13.2 ± 0.7%, 11.9 ± 0.6%) between blastocysts in OEC100 and RN1 + PL/RN2 + EAA + Glu, respectively (Supplementary Table 5). ATP content tended (p = 0.07) to be higher in blastocysts cultured in RN1 + PL/RN2 + EAA + Glu compared to those cultured in OEC100, but this difference was not significant when expressed as the amount of ATP per cell between OEC100 and RN1 + PL/RN2 + EAA + Glu (Supplementary Table 5).

The expression of genes related to metabolism was analyzed in embryos cultured in OEC100 and RN1 + PL/RN2 + EAA + Glu. Relative mRNA levels were based on a control OEC100 level of 1.0. Embryos cultured in RN1 + PL/RN2 + EAA + Glu had elevated expression of *Ldha* and *Hk1*, and reduced expression of *Cpt1b* compared to OEC100 (Fig. 2B). No differences were observed between treatments in the expression of *Acadl*, *Acsl3*, *Cpt2*, *mt-Co2*, *Pdha1*, or *Pdk1*. In addition, western blot analysis was used to determine PDH activity based on the inhibitory phosphorylation level (phosphorylated/total) of PDH^(Ser293)^ in blastocysts after culture in control (OEC100) and reduced nutrient (RN; RN1 + PL/RN2 + EAA + Glu) media (data not shown). Reduced phosphorylation at S293 of PDH E1α subunit in blastocysts derived from RN media indicates increased activity of PDH E1α in this treatment compared to control.

Following embryo transfers, there were no differences between OEC100 and RN1 + PL/RN2 + EAA + Glu in the percentage of pregnant females, or the percentage of females with at least 1 fetus (Table 3). There were also no differences between OEC100 and RN1 + PL/RN2 + EAA + GLU in the percentage of implantations per embryo transferred or the percentage of fetuses per embryos transferred or per implanted embryo (24.7% vs 34.1%, respectively), although an additional 9 fetuses were produced in RN1 + PL/RN2 + EAA + Glu (Table 3). Fetuses produced from embryos cultured in OEC100 or RN1 + PL/RN2 + EAA + GLU did not differ in fetal weight (195.3 ± 9.6 mg, 211.2 ± 8.4 mg, respectively), fetal length (10.8 ± 0.2 mm, 11.0 ± 0.2 mm, respectively), placental weight (158.5 ± 7.8 mg, 162.4 ± 5.6 mg, respectively), or placental diameter (8.2 ± 0.2 mm, 8.6 ± 0.2 mm, respectively) on day 14.5.

**Table 3:** Summary of implantation and fetal development on day 14.5 following surgical embryo transfer of blastocysts that had been cultured in standard (OEC100) or optimized reduced nutrient (RN1 + PL/RN2 + EAA + GLU) culture medium.

Treatment	Total recipients	Total embryos transferred	Pregnant Recipients		Per total embryos transferred		Per implantation
			With ≥ 1 implantation site	With ≥ 1 fetus	Implantations	Fetuses	Fetuses
OEC100	15	120	15 (100%)	10 (66.7%)	81 (67.5%)	20 (16.7%)	20 (24.7%)
RN1 + PL/RN2 + EAA + GLU	15	117	15 (100%)	11 (73.3%)	85 (71.4%)	29 (24.8%)	29 (34.1%)

There were no significant differences (p > 0.05) between treatments.

## Discussion

In the present study we demonstrate that murine embryos are capable of developing from the zygote to the hatching blastocyst stage in culture media with significantly reduced concentrations of nutrients compared to other commonly used media (>13 mM total nutrients) and reports on the composition of oviductal and uterine fluid in the mouse (>21 mM total nutrients; Tables 1 and 2). Reducing nutrient concentrations in culture medium by one half (50% dilution; 6.7 to 7.6 mM total nutrients) did not affect developmental kinetics, blastocyst formation, hatching, the allocation of cells to the TE and ICM, or the ATP content of the embryos. Further reduction of nutrient concentrations delayed development and reduced overall blastocyst formation and hatching, but even in the presence of 25% nutrients (<4 mM total nutrients), approximately 40% of zygotes reached the hatching blastocyst stage. However, if small amounts pyruvate and lactate (equivalent to those in our 50% nutrient medium) were added back to the reduced nutrient (RN, 25% of controls) media, developmental competence could be rescued and resulting blastocysts had increased numbers of ICM cells and more intracellular ATP compared to embryos grown in control conditions. Similarly, embryos produced in the RN medium with elevated (50% of control) concentrations of pyruvate and lactate in the first step medium and EAA and Glu in the second step medium were competent to implant and develop into fetuses at a similar rate as embryos produced in the control medium with 100% nutrient concentrations, despite dramatically different nutrient concentrations and metabolic activity.

Similar to our previous work^17^, analysis of nutrient concentrations in medium following embryo culture in the present study supported our proposed model that embryos only consume a small fraction of the nutrients provided to them. During the second step of culture (48 to 108 h), individual embryos consumed <5% of each nutrient available to them in the control medium. These results are also consistent with other reports on the metabolic activity of murine embryos from other groups using alternative methodologies. For example, the murine blastocyst has been reported to consume approximately 5 pmol of glucose per hour^35,36^. If 10 embryos are cultured in a 20 µl drop of medium for 60 hours, the theoretical uptake of glucose would be 3,000 pmol. If the medium contains 3.0 mM glucose, a 20 µl drop contains 60,000 pmol glucose and the consumption of glucose by 10 embryos would only be ~5% of the total glucose available to the embryo. If blastocysts are cultured with atmospheric oxygen concentrations (20%), glucose consumption is even lower^36^. Obviously, the volume of medium used and the number of embryos present during culture will affect these estimates. However, even a 10-fold reduction in the volume of medium used (10 embryos in 2 vs. 20 µl) or a 10-fold increase in the number of embryos present (100 vs. 10 embryos per 20 µl) would only increase the consumption of glucose by the embryos to ~50% of what is available in media with 3.0 mM glucose. Minimal requirements for glucose during culture are further supported by the excellent developmental rates achieved when murine embryos are cultured for 96 to 120 h in KSOM, which contains only 0.2 mM glucose^13^.

Although reported concentrations (mM) of carbohydrates and amino acids measured in oviduct and uterine fluid from mice^9,37^ and humans^8,38,39^ vary between studies, most culture media, including our control medium, fall within these reported ranges and could be considered “physiological.” In contrast, substrate concentrations in our reduced nutrient media are considerably lower than what has been reported for *in vivo* fluids. However, one must consider that mM concentrations have a volume component (1 mM = 1 mmol per L = 1 nmol per µl) and *in vivo* embryos develop in much smaller volumes of fluid than what occurs in most *in vitro* culture systems^40^. The significantly reduced volume of fluid *in vivo* means the actual amount of any given nutrient (moles) available to the embryo is much lower than what is available to an embryo cultured in a larger volume of medium with the same concentration (moles/L) of nutrients. Of course this is not a simple consideration. It is not well understood if or how the composition of oviductal and uterine fluid is maintained. For example, fluid collected from oviducts containing cumulus oocyte complexes (COC) contains less glucose and more pyruvate than fluid collected from an oviduct lacking COCs^37^. Whether these differences are due to the metabolic activity of the oocytes and cumulus cells or altered secretion of nutrients by the oviductal epithelium in response to the presence of the COC is not known. Limiting the amount of substrate in the embryo’s environment by culturing embryos in reduced volumes of medium, as occurs *in vivo*, or in media with reduced concentrations (mM) of nutrients, as done in the present study, may provide a more physiologically relevant environment rather than simply duplicating *in vivo* substrate concentrations in culture medium formulations.

Pyruvate and lactate (50% of control medium) proved to be the only nutrients necessary to fully rescue embryo development when all other nutrients were present in reduced (25%) concentrations. This is consistent with some of the earliest studies on embryo culture that demonstrated the importance of pyruvate as an energy source for the early mouse embryo. Pyruvate alone supported embryo cleavage to the 2-cell stage in mice^41^, specifically by contributing to the production of ATP by oxidative phosphorylation in the mitochondria^42^. Pyruvate is also a crucial source of energy for human embryos, with glucose unable to compensate for the absence of pyruvate in the medium^43^, despite the fact that more glucose is actually utilized than pyruvate^44^. Although mouse embryos cannot develop when lactate is the only substrate available^45^, the ratio of lactate to pyruvate in the medium effects pyruvate oxidation, as well as intracellular concentrations of NAD + and NADH, and subsequent embryo development^35,46^. In our study, increasing concentrations of pyruvate and lactate proved to be most beneficial when added to step one of our sequential, RN medium, echoing a more critical role for these energy sources during the early cleavage stages^41^. Interestingly, consumption of pyruvate by cleavage stage murine embryos in G1 medium, which is similar to our control medium, has been reported to be ~2 pmol/embryo/h, or ~960 pmol when 10 embryos are cultured for 48 h with reduced oxygen^36^. However, the minimum concentration of pyruvate needed to maintain embryonic development during this period of culture in our study was 0.15 mM (RN + PL) or 3,000 pmol of pyruvate in a 20 µl drop. The apparent requirement for pyruvate in excess of what is needed for metabolism suggests a non-metabolic role for this nutrient, possibly as an antioxidant^33,47^.

We also examined the ability of amino acids to support embryo development in the RN medium containing additional lactate and pyruvate (to 50%). Although murine embryos are capable of developing to the blastocyst stage in medium lacking amino acids^13,48^, the addition of NEAA and/or EAA to culture media improves the developmental competence of embryos compared to embryos grown in the absence of these amino acids^49–51^. Similarly, our analysis of nutrient concentrations at the conclusion of culture indicated that concentrations of several NEAA, including asparagine, aspartate, glycine, and proline, were depleted by>20% by embryos in the RN + PL medium. However, increasing the concentration of NEAA in our reduced nutrient medium from 25% (all NEAA = 0.025 mM) to 50% (all NEAA = 0.05 mM) in one or both steps of culture had no effects on embryo development. Similarly, increasing concentrations of EAA from 25% to 50% (0.125x to 0.25x those found in Minimum Essential Medium, MEM) in the second step medium did not affect embryo development. Biggers *et al*.^13^ evaluated the response of murine embryos to dilutions of the entire complement of NEAA and EAA from 0.5 to 0.03x those concentrations found in MEM and also reported minimal effects on development over the range of concentrations tested in the current study. Together these findings suggest that the amino acid needs of embryos in culture can be met with significantly lower concentrations than those typically used in media or those found in reproductive tract fluids.

Although embryo development appeared to be minimally affected by culture in reduced nutrient conditions when pyruvate and lactate were increased to 50% control concentrations, metabolomic analysis of embryo culture medium was used to determine if development under these conditions involved alterations of the embryo’s metabolic activity. However, results based on relative quantification (% change compared to medium without an embryo) require careful interpretation due to differences in nutrient concentrations in our control and RN + PL media at the initiation of culture^17^. For example, we observed greater production of glutamine by embryos cultured in RN + PL (148%) compared to embryos cultured in the control medium (112%), but there was much less glutamine present in the RN + PL medium (0.25 mM or 6.25 nmol in 25 µl drop) compared to the control medium (1 mM glutamine or 25 nmol in 25 µl drop). Therefore, differences in relative production could be explained by the production of a similar amount of glutamine (~3 nmol) by embryos in both media. Similarly, differences in the proportion of aspartate consumed by the embryos (3% of 2.5 nmol in control vs. 23% of 0.625 nmol in RN + PL) appear to indicate consistent consumption of ~0.1 nmol aspartate by the embryos regardless of the total amount of aspartate present in the medium. Interestingly, alanine metabolism appeared to be differentially regulated in the two culture conditions, with significant production by embryos in the control medium and significant consumption by embryos in RN + PL medium. Production of alanine and glutamine have been reported to be mechanisms used by embryos to detoxify metabolic ammonium^12,34,52,53^. Increased production of alanine, glutamine, and urea by embryos cultured in the control medium may indicate a greater need, or ability, to detoxify ammonium compared to embryos in the RN + PL medium, which only produced glutamine. Alternatively, reduced production of alanine in RN + PL medium could also be a means of conserving pyruvate, since pyruvate is required for the production of alanine from ammonium^34^.

Embryos cultured in RN medium (25% nutrients) exhibited differential expression of several metabolic genes compared to embryos cultured in our control medium. When nutrients were reduced from 50% to 25% of the concentrations in the control medium, expression of *Hk1*, *Ldha*, *Pdha1*, *mt-Co2*, and *Cpt1b* were all decreased, suggesting down regulation of glycolysis (*Hk1*, *Ldha*), the TCA cycle (*Pdha1*), oxidative phosphorylation (*mt-Co2*), and fatty acid oxidation (*Cpt1b*). Why the embryo would down regulate expression of metabolic enzymes in a time of nutrient deprivation is unclear. Interestingly, when the RN medium was supplemented with pyruvate and lactate in step 1 and EAA and Glu in step 2 and development was restored to control levels, expression of *Ldha* and *Hk1* were increased and expression of *Cpt1B* was reduced, which suggests an up regulation of glycolysis, including an enhanced capacity to produce lactate and NAD + from the resulting pyruvate, and a reduced role of fatty acid oxidation, compared to embryos cultured in control medium. However, it is important to note that gene expression does not necessarily reflect protein presence or enzyme activity. Further exploration of the phosphorylation status of PDH E1α protein revealed that PDH activity was increased (decreased inhibitory phosphorylation) in embryos produced in RN1 + PL/RN2 + EAA + Glu media compared to embryos cultured in control medium (data not shown). As PDH E1α activates the PDH complex that converts pyruvate to acetyl-coA_,_ these results would suggest an increase in pyruvate oxidation via the TCA cycle that is not evident in the gene expression data alone^54^.

In summary, this work demonstrates that a sequential media system containing significantly reduced carbohydrate, amino acid, and vitamin concentrations alters embryo metabolism, but still supports the development of embryos with similar viability (cell allocation, ATP content, outgrowth potential, and post-transfer viability) compared to embryos produced in a culture system with higher, more *in vivo*-like, nutrient concentrations. The use of this reduced nutrient medium may provide a novel platform for next generation media development. Further experimentation is underway to evaluate the developmental and metabolic consequences of culturing embryos from other species in media with reduced nutrient concentrations in order to determine which aspects of the current study are unique to the mouse and which aspects are conserved among other mammals. As we continue working to improve embryo culture media, we must be open to challenging the “rules” in order to discover what is truly optimal for the embryo *in vitro*, even when that means using what appear to be non-physiological conditions.

## Supplementary information

Supplementary materials.
