# Supplementary material for: A novel culture medium with reduced nutrient concentrations supports the development and viability of mouse embryos
Source: Sci Rep. 2020 Jun 9;10:9263. doi: 10.1038/s41598-020-66019-4 (PMC7283311; doi:10.1038/s41598-020-66019-4)
Supplement: Supplementary file 1 — Supplementary materials. [file 41598_2020_66019_MOESM1_ESM.pdf]

A novel culture medium with reduced nutrient concentrations supports the development and viability of mouse embryos

Alison F. Ermisch, Jason R. Herrick, Rolando Pasquariello, McKenna C. Dyer, Sarah M. Lyons, Corey D. Broeckling, Sandeep K. Rajput, William B. Schoolcraft, Rebecca L. Krisher

Supplementary Table 1: Summary of primers for target and reference genes used for real-time polymerase chain reaction. *Acadl*, acyl-Coenzyme A dehydrogenase, long-chain; *Acsl3*, acyl-CoA synthetase long-chain family member 3; *Cpt1b*, carnitine palmitoyltransferase 1b; *Cpt2*, carnitine palmitoyltransferase 2; *Hk1*, hexokinase 1; *Ldha*, lactate dehydrogenase A; *mt-Co2*, mitochondrially encoded cytochrome c oxidase II; *Pdha1*, pyruvate dehydrogenase E1 alpha 1; *Pdk1*, pyruvate dehydrogenase kinase 1; *Ppia*, peptidylprolylisomerase A.

**Gene symbol**	**Accession No.**	**Primer Sequence**	
		**Forward (5’ → 3’)**	**Reverse (5’ → 3’)**
Acadl	NM_007381	CTTGCTTGGCATCAACATC	TTCGCAATATAGGGCATGA
Acsl3	NM_001033606	TGCTGAGCTTGTGTGTCTTT	GATCCGATCCATGATTTCC
Cpt1b	NM_009948	TCCAAACGTCACTGCCTAAG	CCAATGTCTCCATGCGGTAATA
Cpt2	NM_009949	TGCTCGCTCAGGATAAACAG	CGGATTGAATGCCATGAA
Hk1	NM_001146100	GAATGCCTCGGAGACTTCAT	TTCAGCAGCTTGACCACATC
Ldha	NM_010699	AGGCTCCCCAGAACAAGATT	TCTCGCCCTTGAGTTTGTCT
mt-Co2	NP_904331	CATCCCAGGCCGACTAAAT	TGGGCATAAAGCTATGGTTAGA
Pdha1	NM_008810	TCACTGCCTATCGAGCACAT	GCCTTTCCCTTTAGCACAAC
Pdk1	NM_172665	TTAGAGGGCTACGGGACAGA	TCGTGGTTGGCTTTGTAATG
Ppia	NM_008907	CGCGTCTCCTTCGAGCGTTTTG	TGTAAAGTCACCACCCTGGCACA

Supplementary Table 2: Experimental design, medium composition of treatments, and evaluated endpoints for each experiment.

Experiment	Step 1 of Culture (0-48 h)		Step 2 of Culture (48-112 h)		Endpoints Evaluated
	Base Nutrients	Modification	Base Nutrients	Modification	
1	100%		100%		Development
	75%		75%		Cell Number and Allocation
	50%		50%		ATP
	25%		25%		Kinetics
					Gene Expression
2	25% (RN)	50% Glucose	25% (RN)	50% Glucose	Development
	25% (RN)	50% EDTA	25% (RN)		Cell Number and Allocation
	25% (RN)	50% Ala-Gln	25% (RN)	50% Ala-Gln	
	25% (RN)	50% Pyruvate + Lactate	25% (RN)	50% Pyruvate + Lactate	
3	25% (RN)		25% (RN)		Development
	25% (RN)	50% Pyruvate + Lactate	25% (RN)		Cell Number and Allocation
	25% (RN)		25% (RN)	50% Pyruvate + Lactate	
	25% (RN)	50% Pyruvate + Lactate	25% (RN)	50% Pyruvate + Lactate	
4	100%		100%		Development
	25% (RN)	50% Pyruvate + Lactate	25% (RN)	50% Pyruvate + Lactate	Cell Number and Allocation
					ATP
					Kinetics
					Outgrowth
					Metabolism
5A	25% (RN)		25% (RN)	50% Pyruvate + Lactate	Development
	25% (RN)	50% Pyruvate + Lactate	25% (RN)	50% Pyruvate + Lactate	Cell Number and Allocation
	25% (RN)	50% NEAA	25% (RN)	50% Pyruvate + Lactate	
	25% (RN)	50% Pyruvate + Lactate + NEAA	25% (RN)	50% Pyruvate + Lactate	
5B	25% (RN)	50% Pyruvate + Lactate	25% (RN)		Development
	25% (RN)	50% Pyruvate + Lactate	25% (RN)	50% Pyruvate + Lactate	Cell Number and Allocation
	25% (RN)	50% Pyruvate + Lactate	25% (RN)	50% NEAA	
	25% (RN)	50% Pyruvate + Lactate	25% (RN)	50% Pyruvate + Lactate + NEAA	
6	25% (RN)	50% Pyruvate + Lactate	25% (RN)		Development
			25% (RN)	50% EAA	Cell Number and Allocation
			25% (RN)	50% Arg	
			25% (RN)	50% Pro	
			25% (RN)	50% Glu	
7	100%		100%		Development
	25% (RN)	50% Pyruvate + Lactate	25% (RN)	50% EAA + Glu	Cell Number and Allocation
					ATP
					Gene Expression
					Embryo Transfer

Supplementary Table 3: Embryo development (per embryo placed into step 2 of culture; cleavage averaged 67.8%) and average number of inner cell mass (ICM; SOX2 positive), trophectoderm (TE; CDX2 positive), and total cells of individual embryos cultured in reduced nutrient (RN) culture medium with the addition of essential amino acids (EAA), arginine (ARG), proline (PRO), or glutamine (GLU) in the second step of culture.

**Treatment**		**Development**				**Cell number (mean ± SEM)**			
**Step one (RN1)**	**Step two (RN2)**	**No. into RN2 treatment**	**No. blastocysts on day 4 (%)**	**No. hatching on day 4 (%)**	**No. hatching on day 5 (%)**	**No.**	**ICM**	**TE**	**Total**
+PL	RN2	78	49 (62.8±5.5)	32 (41.0±5.6)	46 (58.9±5.6)	23	15.0 ± 1.6[xy]	125.3 ± 8.9	140.4 ± 10.3
	+EAA	79	47 (59.5±5.6)	28 (32.9±5.3)	38 (48.1±5.7)	21	15.8 ± 1.4[x]	129.9 ± 11.5	145.6 ± 12.6
	+ARG	78	39 (50.0±5.7)	26 (33.3±5.4)	38 (48.7±5.7)	21	11.3 ± 1.4[y]	117.4 ± 7.5	128.7 ± 8.3
	+PRO	79	40 (50.6±5.7)	23 (29.1±5.1)	39 (49.4±5.7)	24	11.2 ± 1.3[y]	105.5 ± 8.1	116.7 ± 9.1
	+GLU	80	53 (66.3±5.3)	34 (42.5±5.6)	50 (63±5.4)	32	14.0 ± 1.3[xy]	121.1 ± 5.6	135.2 ± 6.5

[xy]Different superscripts indicate trends ($p=0.09$) between treatments within a given endpoint

Supplementary Table 4: Embryo development (per total zygotes) following culture in control (OEC100) and reduced nutrient culture medium (RN1+PL/RN2+EAA+GLU).

Treatment	Development					
	No. fertilized	No. cleaved (%)	No. blastocysts on day 4 (%)	No. hatching on day 4 (%)	No. hatching on day 5 (%)
OEC100	194	170 (87.6±2.3)	131 (67.5±3.4)	103 (53.1±3.6)^{x}	127 (65.5±3.4)
RN1+PL/ RN2+EAA+GLU	206	185 (89.3±2.2)	137 (66.5±3.3)	117 (56.8±3.5)^{y}	136 (66.0±3.3)

^{xy}Different superscripts indicate trends (p=0.09) between treatments within a given endpoint

Supplementary Table 5: Number of inner cell mass (ICM; SOX2 positive), trophectoderm (TE; CDX2 positive), and total cells, and average ATP concentration, of individual hatching blastocysts cultured in control (OEC100) and reduced nutrient culture medium (RN1+PL/RN2+EAA+GLU) culture medium.

Treatment	Cell number (mean ± SEM)				ATP		
	No. of embryos	ICM	TE	Total	No. of embryos	Concentration (pmol/embryo)	Concentration (pmol x 10^{-3}/cell)
OEC 100	45	18.3 ± 1.3	123.6 ± 6.3[a]	142.9 ± 7.2[a]	19	0.35 ± 0.04[x]	2.5 ± 0.3
RN1+PL/ RN2+EAA+GLU	46	19.9 ± 1.1	148.8 ± 6.3[b]	168.7 ± 7.0[b]	17	0.46 ± 0.04[y]	2.7 ± 0.2

[ab]Different superscripts indicate significant differences (p≤0.01) between treatments within a given endpoint

[xy]Different superscripts indicate trends (p=0.09) between treatments within a given endpoint

Supplementary Figure 1

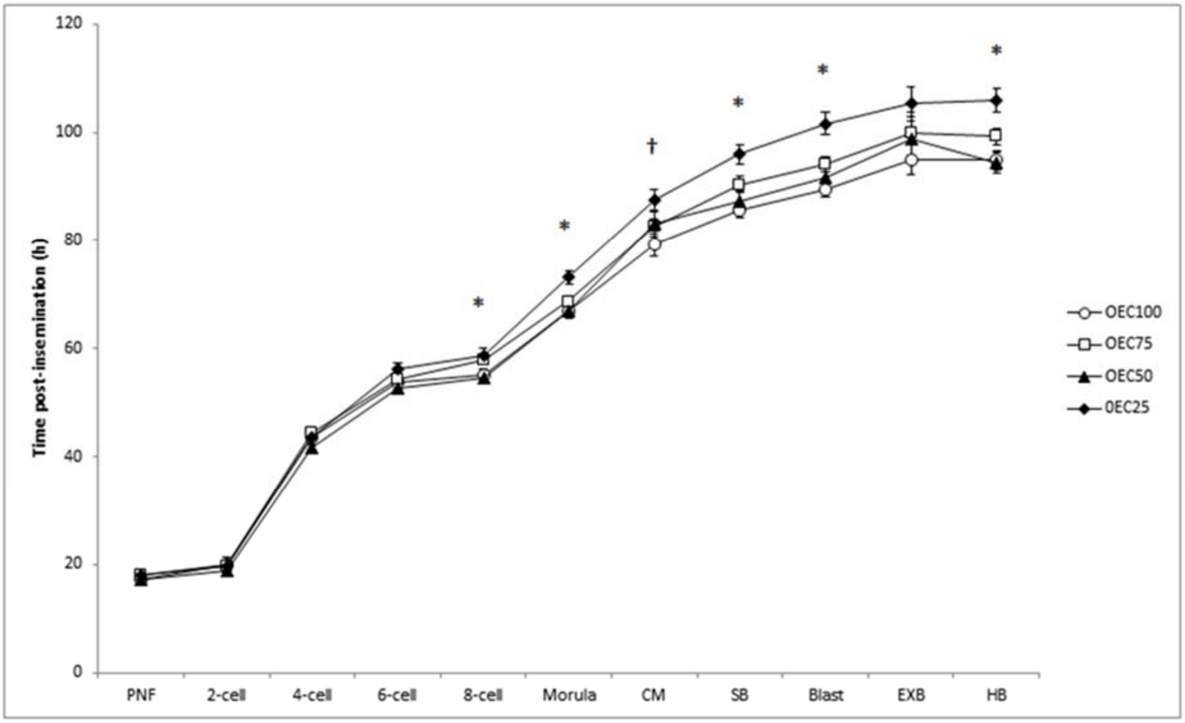

Supplementary Figure 1: Kinetics of individually cultured embryos in control (OEC100) or reduced nutrient (OEC75, OEC50, OEC25) culture medium. Times are reported in hours post insemination. PNF, pro nuclei fade; CM, compact morula; SB, start blastoceol; Blast, blastocyst; EXB, expanded blastocyst; HB, hatching blastocyst. Data are presented as the mean ± s.e.m. Superscripts indicate significant differences (*; $p \leq 0.01$) or trends (†; $p=0.06$) between OEC100 and OEC25 treatments within a given endpoint.

Supplementary Figure 2

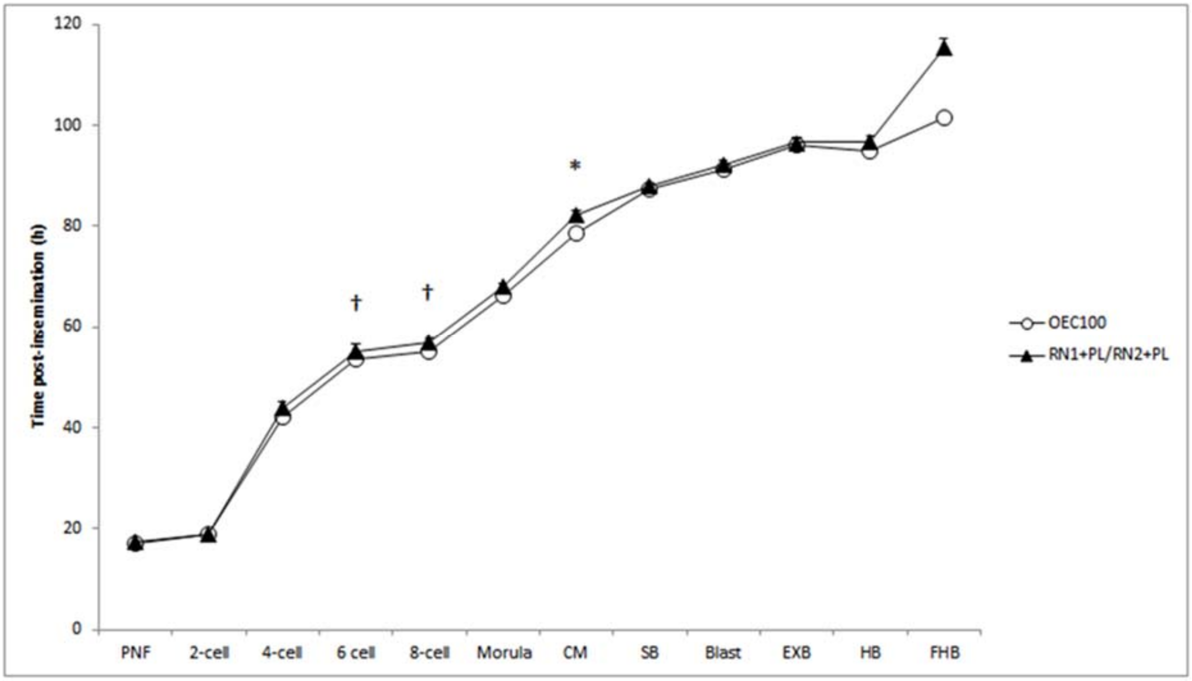

Supplementary Figure 2: Kinetics of individually cultured embryos in control (OEC100) or reduced nutrient (RN1+PL/RN2+PL) culture medium. Times are reported in hours post insemination. PNF, pro nuclei fade; CM, compact morula; SB, start blastoceol; Blast, blastocyst; EXB, expanded blastocyst; HB, hatching blasstocyst; FHB, fully hatched blastocyst. Data are presented as the mean ± s.e.m. Different superscripts indicate significant differences (*; p=0.02 or trends (†; p=0.09) between treatments within a given endpoint.
